# Vitamin B_12_ and Folic Acid Imbalance Modifies NK Cytotoxicity, Lymphocytes B and Lymphoprolipheration in Aged Rats

**DOI:** 10.3390/nu5124836

**Published:** 2013-11-26

**Authors:** Teresa Partearroyo, Natalia Úbeda, Ana Montero, María Achón, Gregorio Varela-Moreiras

**Affiliations:** Department of Pharmaceutical and Health Sciences, Faculty of Pharmacy, CEU San Pablo University, Boadilla del Monte, Madrid 28668, Spain; E-Mails: t.partearroyo@ceu.es (T.P.); nubeda@ceu.es (N.U.); amontero.fcex@ceu.es (A.M.); achontu@ceu.es (M.A.)

**Keywords:** folic acid, vitamin B_12_, rats, aging, immunology

## Abstract

Different vitamin B_12_ and folic acid concentrations could exacerbate the immune response. The aim was to evaluate different dietary folic acid and vitamin B_12_ levels on the immune response in aged rats. Male Sprague Dawley aged rats were assigned to three folic acid groups (deficient, control, supplemented) each in absence of vitamin B_12_ for 30 days. Several parameters of innate and acquired immune responses were measured. Serum and hepatic folate levels increased according to folic acid dietary level, while vitamin B_12_ levels decreased. There was a significant decrease in natural killer cell-mediated cytotoxicity in the spleen for the vitamin B_12_ deficient diet and folic acid control diet groups. Significant changes in CD45 lymphocyte subsets were also observed according to dietary imbalance. Lymphoproliferative response to concanavalin A and phytohemagglutinin did not differ significantly between groups. The spleen response to lipopolysaccharide increased significantly, but was unmodified for the other organs. An imbalance between dietary vitamin B_12_ and folic acid concentrations alters some immunological parameters in aged rats. Therefore, the ratio between folate and vitamin B_12_ could be as important as their absolute dietary concentrations.

## 1. Introduction

From 1998 the United States of America (USA) and Canada implemented a nutrition policy of mandatory fortification with the vitamin folic acid (FA) in flour and grain products. This nutrition policy was established following a proposal by the Food and Drug Administration [1], aimed at preventing neural tube defects (NTD). The fortification program has achieved considerable success in its main goal, since the incidence of NTD in the USA has declined in a range of 19%–27% [2,3,4,5]. In Spain where no mandatory fortification policy exists, we have shown that overages are a current practice in FA fortified breakfast cereals and milk products, and total folate values were higher than those declared by manufacturers in most cases [6,7]. The most well known adverse effect of supplementation and for food fortification with FA is the masking of the diagnosis of B_12_ deficiency, because megaloblastic anaemia caused by cobalamin deficiency can be reversed, but not the potential long-term neurological effects [8]. Morris *et al.* [9] observed that low B_12_ status and high serum folate levels were associated with cognitive impairment and anaemia in the elderly. By contrast, adequate B_12_ status and high serum folate levels were associated with protection against cognitive impairment. This has led to a controversy about the effects of supplementation and/or fortification with FA in subjects with vitamin B_12_ deficiency.

Moreover, the two vitamins play a potentially important role in immune function [10]; in fact, folate deficiency could lead to many clinical alterations including reduced immune function [11]. More specifically, folate deficiency in cultured phytohaemaglutinin (PHA)-activated human T lymphocytes, is able to reduce T lymphocyte proliferation, but also to induce apoptosis and increase the CD4^+^ to CD8^+^ ratio due to a marked reduction of CD8^+^ cell proliferation [12], which may lead to a lower resistance to infections [11]. Therefore, another potential effect of supplementation with FA could be the improvement in immune function. Field *et al.* [13], by supplementing rats with additional folate for three weeks, observed that the proliferative response to mitogens, the distribution of T cells in mesenteric lymph node and age-related changes in cytokine production in the spleen were all improved.

It is now proposed that dietary folate requirements may be higher during the aging process, to support and protect immune function, since immunity deteriorates with age [14,15]. Decreased T cell memory and exhaustion of the naive T cell population with involution of the thymus are commonly observed in the elderly [16]. Troen *et al.* [17] found in postmenopausal women a “U-shaped” relation between total folate intake and natural killer cells (NK) cytotoxicity. They also showed that unmetabolized FA in plasma is associated with decreased NK cytotoxicity. Approximately 40% of older adults in the USA have unmetabolized serum folic acid that persists after fasting [18]. Thus, fortification or supplementation with FA may suppress the NK function, which is critical for normal immune function. Tamura *et al.* [19] have suggested that vitamin B_12_ may play an important role in cellular immunity, mainly affecting the CD8^+^ cells and the NK cell system, which suggests effects on cytotoxic cells.

In consequence, the present study aimed at developing an aged animal model in order to evaluate some critical immunological parameters related to both FA and B_12_ status in the aging process. For this purpose, the influence of FA status is evaluated using different dietary FA levels, from deficiency to a supplemented state under experimental B_12_ induced deficiency.

## 2. Material and Methods

### 2.1. Animals and Diets

Twenty-month-old OFA male (Sprague Dawley) rats were obtained from Charles River Laboratories (Barcelona, Spain). Animals were maintained on a 12:12 h dark/light cycle, under temperature and humidity controlled conditions at the Animal Care Unit (Universidad CEU San Pablo, Madrid, Spain). All animal use and handling were performed following the European Union Normative (2003/65/CE) and were reviewed and ethically approved by the Animal Experimentation Committee of the Universidad CEU San Pablo.

### 2.2. Dietary Treatment

The animals were randomly assigned to be fed either a vitamin B_12_ deficient diet (*n* = 27) or a control diet (*n* = 8). After receiving the assigned diet for eight weeks, they were divided in four different groups on the basis of the experimental diet used, that were adjusted to rat requirements and based on a pure amino-acid diet (Dyets, Bethlehem, PA, USA) [20] modifying the FA and B_12_ content as follows: B_12_ and FA deficient diet (Group D_B12_D_FA_, 0 μg B_12_/kg diet and 0 mg FA/kg diet, *n* = 9), B_12_ deficient diet and FA control diet (Group D_B12_C_FA_, 0 μg B_12_/kg diet and 2 mg FA/kg diet, *n* = 9), B_12_ deficient diet and FA supplemented diet (Group D_B12_S_FA_, 0 μg B_12_/kg diet and 8 mg FA/kg diet, *n* = 9), and control diet (Group C_B12_C_FA_, 50 μg de B_12_/kg diet and 2 mg FA/kg diet, *n* = 8). Rats were fed *ad libitum* their respective diets for 30 days.

### 2.3. Sacrifice and Tissue Handling

Animals were sacrificed by decapitation; liver, spleen, thymus and axillary nodes were quickly removed. Whole blood was collected from all rats and serum and plasma were separated by centrifugation and kept at −80 °C until further analyses.

### 2.4. Serum Folate

Serum folate levels were analyzed by a microbiological method using *Lactobacillus casei* (ATCC 7469) (American Type Culture Collection), as previously reported by Horne and Tamura [21,22].

### 2.5. Hepatic Folate

Folate content in the rat liver was assessed by a microbiological method above described for serum folate determination. Previously, the livers were prepared through extraction and enzyme treatments carried according to the described trienzyme extraction method [23].

### 2.6. Serum Vitamin B_12_

Serum vitamin B_12_ levels were determinated by commercial kit (Immundiagnostik AG, Bensheim, Germany). The serum was pretreated and diluted in a buffer and then the samples were transferred into a 96 well-plate and then coated with *Lactobacillus delbrueckii* (ATCC 7830). The vitamin B_12_ quantification depends on the growth of the organism after incubation at 37 °C for 44–48 h. The growth of *Lactobacillus* turbidimetrically is determined at 610–630 nm. The vitamin B_12_ concentration is directly proportional to the turbidity.

### 2.7. Isolation of Rat Blood Neutrophils and Lymphocytes

Peripheral blood lymphocytes were obtained following a previously described method [24], by gradient sedimentation using a 1.077 density Hystopaque (Sigma-Aldrich, Spain) for lymphocytes. Cells from both interfaces were washed twice in phosphate-buffered saline solution. Pelleted cells were resuspended and diluted to a final concentration of 2 × 10^5^ lymphocytes/mL medium. The lymphocytes were then incubated with monoclonal antibodies at 4 °C for 25 min in the dark. The following lymphocyte subsets were evaluated by flow cytometry: CD3, CD4, CD8, CD45RA and CD161. The control samples were incubated with purified phycoerythrin-labeled mouse immunoglobulin G1, fluorescein isothiocyanate-labeled mouse immunoglobulin G2 and *allophycocyanin*-labeled mouse immunoglobulin M. The fluorescence of the subsets was analyzed with a Facstar Plus dual-laser cytometer (Becton Dickinson, Sunnyvale, CA, USA) [25].

### 2.8. Lymphoproliferation

A previously described method was used [24,26,27]. Two hundred microliters of lymphocyte suspension, adjusted to 10^6^ cells/mL in complete medium (RPMI-1640, PAA; plus 10% fetal bovine serum, Gibco; plus 1% gentamicin, PAA, Pasching, Austria), was cultured in plates with concanavalin A (Sigma-Aldrich, Spain) (Con A, 5 μg/mL in well), lipopolysaccharide (Sigma-Aldrich, Spain) (LPS, *Escherichia coli*, 055:B5, 5 μL/mL in well) and phytohaemagglutinin (Sigma-Aldrich, Spain) (PHA, 50 μg/mL in well) as stimulated samples or in complete medium as non-stimulated samples. After 48 h of incubation, 5 μ Ci [^3^H]thymidine (Biolink 2000, Spain) was added to each well and cells were harvested. Results were expressed as the “stimulation index”, which is the percentage of stimulation in response to Con A, LPS or PHA, with non-stimulated values (counts per minute) being 100%.

### 2.9. Cytotoxicity Assay

Murine lymphoma YAC-1 cells were used as targets in the NK activity assay [26]. An enzymatic colorimetric assay was used for cytolysis measurements of target cells (Cytotox 96 TM Promega, Madison, WI, USA) based on determination of lactate deshydrogenase using tetrazolim salts. Cells were cultured at an effector/target rate of 10/1 and incubated for 4 h. Then lactate deshydrogenase enzymatic activity was measured in 50 μL/well of supernatants by addition of the enzyme substrate and absorbance spectrophotometrically recorded at 490 nm. Results were expressed as percentage of lysis of target cells.

### 2.10. Interleukins (IL), Granulate-Macrophage Stimulating Factor Precursor (GM-CSF), Interferon Gamma (IFN-*γ*), Tumor Necrosis Factor Alpha (TNF-*α*) Levels

IL-1A, IL-1B, IL-4, IL-12, GM-CSF, IFN-γ, TNF-α levels were determined on culture supernatants of lymphocyte after a 48 h incubation with the mitogen Con A, LPS and PHA, following a method previously described by Carrasco *et al.* [28] and measured using the multiplex antibody kits for the Luminex^®^ system. All tests were determined by Cytokine 10-Plex rat panel (Invitrogen, Germany) for the simultaneous determination of IL-1a, IL-1b, IL-4, IL-12, TNF-α and IFN-γ in cell culture. The results were expressed as picograms per milliliter.

### 2.11. Statistical Analysis

Parametric data were statistically analyzed by a one way Analysis of variance (ANOVA). When ANOVA resulted in differences, multiple comparisons between means were studied by Bonferroni tests. Values are expressed as mean (95% confidence interval). Differences were considered significant at *p* < 0.05. Variables were tested for normality using a Kolmogorov-Smirnov test (SPSS 15.0, SPSS Inc.: Chicago, IL, USA).

## 3. Results

To demonstrate the usefulness of our experimental model, we determined serum and hepatic FA and serum vitamin B_12_ levels after dietary treatment. Serum vitamin B_12_ concentration was significantly decreased in the vitamin B_12_ deficient diet groups (*p* < 0.001) when compared to control animals (Table 1). Serum and hepatic folate levels were increased, as expected, in accordance with the dietary vitamin supplementation level (*p* < 0.05 and *p* < 0.001), as also shown in Table 1.

**Table 1:** Serum and hepatic folate and serum vitamin B_12_ concentrations.

Group	Serum Folate (ng/mL)	Liver Folate (μg/g liver)	Serum Vitamin B_12_ (ng/L)
C_B12_C_FA_	38.6	18.3	1203.4
*n* = 8	(34.1 to 43.1)	(14.7 to 22.0)	(1036.6 to 1370.3)
D_B12_D_F__A_	4.6 ***^,###^	7.6 **^,#^	744.4 ***
*n* = 9	(4.0 to 5.2)	(4.9 to 10.2)	(663.0 to 825.7)
D_B12_C_FA_	62.5 *	16.1	906.8 ***
*n* = 9	(54.2 to 70.8)	(11.0 to 21.2)	(845.7 to 967.9)
D_B12_S_FA_	114.5 ***^,###^	29.6 **^,##^	807.6 ***
*n* = 9	(92.8 to 136.3)	(23.6 to 35.7)	(738.1 to 877.0)

Serum and hepatic folate and serum vitamin B_12_ concentrations in male rats fed B_12_ and FA deficient diets (Group D_B12_D_FA_, 0 μg B_12_/kg diet and 0 mg FA/kg diet), B_12_ deficient diet and FA control diet (Group D_B12_C_FA_, 0 μg B_12_/kg diet and 2 mg FA/kg diet), B_12_ deficient diet and FA supplemented diet (Group D_B12_S_FA_, 0 μg B_12_/kg diet and 8 mg FA/kg diet), and control diet (Group C_B12_C_FA_, 50 μg B_12_/kg diet and 2 mg FA/kg diet). Values are means (95% confidence interval per group). * *p* < 0.05 *vs.* group C_B12_C_FA_; ** *p* < 0.01 *vs.* group C_B12_C_FA_, *** *p* < 0.001 *vs.* group C_B12_C_FA_; ^#^ *p* < 0.05 *vs.* group D_B12_C_FA_; ^##^ *p* < 0.01 D_B12_C_FA_; ^###^ *p* < 0.001 D_B12_C_FA_. (Bonferroni’s test).

**Figure 1:**
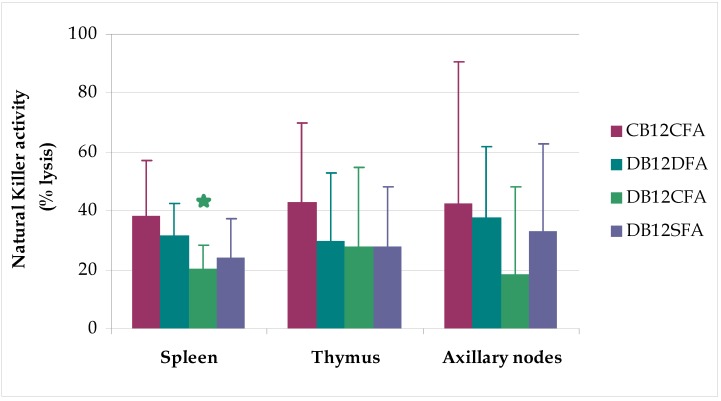
Natural killer activity in spleen, thymus and axillary nodes. Natural killer activity in male rats fed B_12_ and FA deficient diets (*n* = 4, Group D_B12_D_FA_, 0 μg B_12_/kg diet and 0 mg FA/kg diet), B_12_ deficient diet and FA control diet (*n* = 3, Group D_B12_C_FA_, 0 μg B_12_/kg diet and 2 mg FA/kg diet), B_12_ deficient diet and FA supplemented diet (*n* = 4, Group D_B12_S_FA_, 0 μg B_12_/kg diet and 8 mg FA/kg diet), and control diet (*n* = 3, Group C_B12_C_FA_, 50 μg B_12_/kg diet and 2 mg FA/kg diet). Values are means (95% confidence interval per group) * *p* < 0.05 *vs.* group C_B12_C_FA_ (Bonferroni’s test).

There was a significant decrease in the NK cytotoxicity (Figure 1) in the spleen, in the DB12CFA group respective to the control group (*p* < 0.05) but neither in the thymus nor the axillary nodes. Furthermore, we observed a significant decrease in the B lymphocyte subsets in groups DB12CFA and DB12SFA (*p* < 0.05) but not for lymphocyte subsets helper T cells, cytotoxic-supressor T cells, mature T cells and natural killer cells (Table 2). Consequently, it seems that B_12_ deficiency decreases both B-cell diversity and the NK activity.

**Table 2:** Lymphocyte subsets in peripheral blood lymphocytes (%).

Group	Helper Tcells	Cytotoxic-Supressor T cells	CD4/CD8	Mature Tcells	B lymphocytes	Natural Killer Cells
C_B12_C_FA_	24.6	9.6	2.7	35.5	18.2	15.6
*n* = 7	(18.1 to 31.1)	(6.7 to 12.5)	(2.0 to 3.3)	(26.9 to 44.1)	(12.3 to 24.0)	(9.82 to 21.4)
D_B12_D_FA_	20.6	10.0	2.2	32.2	12.5	15.7
*n* = 8	(13.7 to 27.4)	(6.6 to 13.3)	(1.5 to 2.8)	(22.8 to 41.6)	(6.2 to 18.9)	(4.8 to 26.5)
D_B12_C_FA_	26.5	10.4	2.6	38.4	7.3 *	5.0
*n* = 7	(20.1 to 32.8)	(7.5 to 13.4)	(2.1 to 3.1)	(29.2 to 47.5)	(2.1 to 12.5)	(1.7 to 8.4)
D_B12_S_FA_	21.6	7.5	3.5	30.4	8.9 *	28.0
*n* = 8	(18.3 to 24.9)	(4.5 to 10.6)	(2.0 to 5.0)	(24.3 to 36.4)	(4.0 to 13.7)	(11.9 to 44.1)

Helper T cells (CD4), cytotoxic-supressor T cells (CD8), CD4/CD8 ratio, mature T cells (CD3), B lymphocytes (CD45RA), and natural killer cells (CD161) in male rats fed B_12_ and FA deficient diets (Group D_B12_D_FA_, 0 μg B_12_/kg diet and 0 mg FA/kg diet), B_12_ deficient diet and FA control diet (Group D_B12_C_FA_, 0 μg B_12_/kg diet and 2 mg FA/kg diet), B_12_ deficient diet and FA supplemented diet (Group D_B12_S_FA_, 0 μg B_12_/kg diet and 8 mg FA/kg diet), and control diet (Group C_B12_C_FA_, 50 μg B_12_/kg diet and 2 mg FA/kg diet). Values are means (95% confidence interval per group). * *p* < 0.05 *vs.* group C_B12_C_FA_ (Bonferroni’s test).

Lymphoproliferation induced by mitogenic agents (Table 3) showed the response to ConA for the three organs (spleen, thymus and axillary nodes). As it is shown, it did not differ significantly between groups, and the same phenomenon was observed for PHA. However, the response to LPS in the spleen increased significantly in the group D_B12_S_FA_, but not in the other organs. Therefore, the spleen seems to be more sensitive to changes in the presence of endotoxins when compared to the thymus or the axillary nodes.

**Table 3:** Lymphoproliferative response to concanavalin A (Con A), lipopolysaccharide (LPS) and phytohemagglutinin (PHA) in spleen, thymus and axillary nodes (%).

**Organ**	**Mitogen**	**C_B12_C_FA_**	**D_B12_D_FA_**	**D_B12_C_FA_**	**D_B12_S_FA_**
		***n* = 8**	***n* = 9**	***n* = 9**	***n* = 9**
**Spleen**	**Con A**	138.1	168.0	185.4	146.3
		(87.4 to 188.8)	(111.1 to 224.9)	(173.6 to 197.2)	(110.7 to 181.9)
	**LPS**	140.0	178.1	223.0	207.4 *
		(98.3 to 181.7)	(129.1 to 227.2)	(142.5 to 303.5)	(133.3 to 281.6)
	**PHA**	177.1	164.1	270.8	180.6
		(90.1 to 264.2)	(130.9 to 197.4)	(156.8 to 384.8)	(97.0 to 264.1)
**Organ**	**Mitogen**	**C_B12_C_FA_**	**D_B12_D_FA_**	**D_B12_C_FA_**	**D_B12_S_FA_**
		***n* = 8**	***n* = 9**	***n* = 8**	***n* = 9**
**Thymus**	**Con A**	111.3	161.1	151.3	125.2
		(58.9 to 163.7)	(126.7 to 195.5)	(102.5 to 200.1)	(116.9 to 133.5)
	**LPS**	152.9	148.6	141.0	157.8
		(59.2 to 246.6)	(108.8 to 188.3)	(92.7 to 189.3)	(131.8 to 183.8)
	**PHA**	161.3	149.9	204.9	154.8
		(53.3 to 269.3)	(124.7 to 175.0)	(132.9 to 276.8)	(121.2 to 188.4)
**Organ**	**Mitogen**	**C_B12_C_FA_**	**D_B12_D_FA_**	**D_B12_C_FA_**	**D_B12_S_FA_**
		***n* = 6**	***n* = 8**	***n* = 7**	***n* = 7**
**Axillary nodes**	**Con A**	115.6	152.0	171.0	131.5
		(102.2 to 129.0)	(74.1 to 229.9)	(42.0 to 300.0)	(108.9 to 154.1)
	**LPS**	110.2	191.2	211.7	139.0
		(63.8 to 156.6)	(109.8 to 272.5)	(27.7 to 395.6)	(105.9 to 172.1)
	**PHA**	145.2	190.2	181.0	149.5
		(125.0 to 165.4)	(148.3 to 232.0)	(43.6 to 318.4)	(81.0 to 218.0)

Lymphoproliferative response to Con A, LPS and PHA in spleen, thymus and axillary nodes in male rats fed B_12_ and FA deficient dies (Group D_B12_D_FA_, 0 μg B_12_/kg diet and 0 mg FA/kg diet), B_12_ deficient diet and FA control diet (Group D_B12_C_FA_, 0 μg B_12_/kg diet and 2 mg FA/kg diet), B_12_ deficient diet and FA supplemented diet (Group D_B12_S_FA_, 0 μg B_12_/kg diet and 8 mg FA/kg diet), and control diet (Group C_B12_C_FA_, 50 μg B_12_/kg diet and 2 mg FA/kg diet). Values are means (95% confidence interval per group). * *p* < 0.05 *vs.* group C_B12_C_FA_ (Bonferroni’s test).

IL-1a, IL-1b, IL-4, IL-12, GM-CSF, IFN-γ, TNF-α, TNF-α/IL-4 concentrations are reflected in Table 4. The concentrations of these interleukins are not modified by the effect of cyanocobalamin deficiency or by the FA level. The proportion of TNF-α to IL-4 is reflected in Table 4. As shown, there is no variation under the four experimental conditions tested.

**Table 4:** Interleukins, granulate-macrophage stimulating factor precursor (GM-CSF), interferon gamma (IFN-γ), tumor necrosis factor alpha (TNF-α) levels in basal conditions and in presence of concanavalin A (ConA), lipopolysaccharide (LPS) and phytohemagglutinin (PHA).

Cytokines	Mitogen	C_B12_C_FA_ *n =* 5	D_B12_D_FA_ *n =* 5	D_B12_C_FA_ *n =* 5	D_B12_S_FA_ *n =* 5
		*n =* 5	*n =* 5	*n =* 5	*n =* 5
**IL-1a (pg/mL)**	**Basal**	54.9 (53.9 *–*55.9)	54.6 (46.7 *–*62.6)	53.8 (49.6 *–*57.9)	53.5 (50.7 *–*56.3)
	**Con A**	56.0 (43.3 *–*68.7)	54.3 (44.7 *–*63.8)	53.3 (51.9 *–*54.8)	58.0 (46.2 *–*69.8)
	**LPS**	64.3 (54.3 *–*74.4)	58.0 (55.5 *–*60.5)	62.0 (48.9 *–*75.1)	62.6 (56.8 *–*68.5)
	**PHA**	58.0 (54.6 *–*61.4)	59.1 (53.8 *–*64.5)	55.8 (50.7 *–*60.9)	54.3 (52.8 *–*55.8)
**IL-1b (pg/mL)**	**Basal**	13.8 (3.0 *–*24.7)	15.0 (6.9 *–*23.1)	18.3 (8.8 *–*27.7)	13.1 (10.7 *–*15.5)
	**Con A**	9.7 (2.1 *–*17.3)	9.1 (−6.1 *–*24.3)	14.2 (5.3 *–*23.1)	12.1 (8.5 *–*15.6)
	**LPS**	20.5 (17.1–23.9)	17.3 (17.3–17.3)	26.6 (9.6–43.6)	18.5 (11.3–25.8)
	**PHA**	18.0 (3.05–33.0)	18.1 (6.4–29.8)	18.5 (12.0–25.1)	12.1 (8.9–15.4)
**IL-4 (pg/mL)**	**Basal**	4.39 (4.35–4.42)	4.40 (4.34–4.45)	4.39 (4.35–4.42)	4.38 (4.36–4.41)
	**Con A**	4.42 (4.18–4.67)	4.39 (4.32–4.45)	4.38 (4.33–4.43)	4.39 (4.35–4.42)
	**LPS**	4.39 (4.34–4.45)	4.39 (4.34–4.45)	4.39 (4.34–4.45)	4.38 (4.35–4.41)
	**PHA**	4.43 (4.41–4.45)	4.41 (4.41–4.41)	4.41 (4.41–4.41)	4.31 (4.13–4.49)
**IL-12 (pg/mL)**	**Basal**	31.5 (−2.8–65.7)	24.8 (9.3–40.3)	27.4 (9.5–45.2)	35.5 (27.3–43.7)
	**Con A**	18.9 (−67.5–105.3)	28.2 (18.1–38.3)	29.1 (14.6–43.6)	18.7 (27.3–36.7)
	**LPS**	54.1 (33.0–75.2)	30.8 (18.3–43.4)	61.1 (−4.5–126.7)	54.3 (27.2–81.4)
	**PHA**	29.4 (−8.2–66.9)	29.1 (14.6–43.6)	41.2 (12.4–69.9)	32.5 (22.8–42.2)
**GM-CSF (pg/mL)**	**Basal**	12.9 (11.9–14.0)	13.6 (12.6–14.5)	13.3 (13.3–13.3)	12.5 (11.6–13.4)
	**Con A**	13.9 (6.1–21.7)	13.5 (12.4–14.6)	12.4 (10.5–14.3)	12.9 (11.9–14.0)
	**LPS**	13.7 (11.9–15.4)	13.7 (11.9–15.4)	12.8 (11.0–14.7)	13.6 (12.6–14.5)
	**PHA**	13.7 (11.9–15.4)	13.7 (11.9–15.4)	13.3 (13.3–13.3)	13.3 (13.3–13.3)
**IFN-γ (pg/mL)**	**Basal**	3.0 (1.3–4.7)	2.0 (−0.2–4.1)	2.7 (0.7–4.6)	3.0 (0.7–5.2)
	**Con A**	2.3 (−6.0–10.8)	1.7 (1.7–1.7)	1.2 (−1.0–3.3)	2.98 (0.6–5.4)
	**LPS**	3.4 (−0.3–7.1)	2.1 (0.2–4.0)	3.0 (−0.2–6.2)	4.2 (−0.0–8.4)
	**PHA**	2.8 (1.9–3.7)	2.1 (0.2–4.03)	3.0 (1.4–4.6)	2.7 (2.0–3.5)
**TNF-α (pg/mL)**	**Basal**	13.6 (−1.0–28.2)	13.9 (0.1–27.7)	11.9 (5.7–18.2)	17.6 (11.6–23.5)
	**Con A**	13.9 (1.5–29.1)	9.9 (3.5–16.3)	11.0 (0.2–21.9)	15.5 (5.7–25.3)
	**LPS**	80.9 (66.1–95.7)	56.0 (−23.8–135.9)	67.3 (−11.8–146.3)	64.4 (41.4–87.3)
	**PHA**	27.0 (−11.8–65.9)	17.03 (0.4–33.6)	44.4 (0.7–88.1)	34.9 (14.2–55.6)
**TNF-α/IL-4**	**Basal**	3.1 (−0.2–6.4)	3.2 (−0.0–6.4)	2.7 (1.3–4.2)	4.0 (2.7–5.3)
	**Con A**	3.1 (−1.2–7.5)	2.1 (1.2–2.9)	2.7 (1.2–4.3)	4.2 (1.8–6.7)
	**LPS**	19.7 (15.3–24.1)	13.2 (3.7–22.8)	13.2 (1.6–24.7)	14.7 (9.5–19.9)
	**PHA**	8.4 (−0.2–16.9)	3.9 (0.1–7.6)	10.1 (0.2–20.0)	8.1 (3.3–13.0)

IL-1a, IL-1b, IL-4, IL-12, GM-CSF, IFN-γ, TNF-α and TNF-α/IL-4 in male rats fed B_12_ and FA deficient diets (Group D_B12_D_FA_, 0 μg B_12_/kg diet and 0 mg FA/kg diet), B_12_ deficient diet and FA control diet (Group D_B12_C_FA_, 0 μg B_12_/kg diet and 2 mg FA/kg diet), B_12_ deficient diet and FA supplemented diet (Group D_B12_S_FA_, 0 μg B_12_/kg diet and 8 mg FA/kg diet), and control diet (Group C_B12_C_FA_, 50 μg B_12_/kg diet and 2 mg FA/kg diet). Values are means (95% confidence interval per group).

## 4. Discussion

In the present study, we examined the effects of an imbalance between vitamin B_12_ and FA dietary concentration on NK cytotoxicity, B lymphocytes and lymphoprolipheration in aged rats. A marked decrease in the spleen NK activity was observed in the D_B12_C_FA_ group, although this effect was not statistically significant in the thymus or axillary nodes.

Although just few studies have examined the relationship between immune response and both B_12_ and FA, it has been observed in humans that either a deficiency or an excessive dietary folate appear to be related to an alteration of the immune response, as determined by NK cytotoxicity. In this intervention study, 105 healthy women were therefore exposed to the mandatory fortification with FA in flour and grain products: women whose diets were low in folate and consumed supplements increased the NK cytotoxicity. However, women who consumed a folate-rich diet plus FA supplements had reduced NK cytotoxicity compared with those consuming a low-folate diet and no supplements. In another study by contrast, there was not correlation between plasma total folate and cytotoxicity of NK cells [29].

In experimental animal models, moderate folate deficiency appears not to affect the NK-mediated cytotoxicity, in contrast to that observed in severe folate deficiency [30]. It has been also reported that decreased NK mediated cytotoxicity in aged animals was reverted when feeding them with diets supplemented with antioxidants [31,32].

Following this same pattern, our experiments also revealed that lymphocyte subsets (T-helper cells, cytotoxic or suppressor cells, mature T cells, B cells and Natural Killers) were lower for B cells in D_B12_C_FA_ and D_B12_S_FA_ groups, which demonstrates the importance of an equilibrium between folate and B_12_ intake. Gibson *et al.* [33] have recently observed that B-cell diversity may dramatically decrease with age and may have important implications for the immune health of elderly people. More studies are necessary to determine the possibility of a causal link between B_12_ deficiency and loss of B-cells diversity, and whether B-cells could be improved by restoring normal levels of B_12_ and/or folate.

It should be noted that the best-known contribution of B-cells in the immune system is the production of antibodies. These cells are highly effective as antigen-presenting cells and essential for the development of T-cells [34]. There is also evidence to support the role of B-cells as immune regulators, because they are able to secrete IL-10 which could prevent inadequate stimulation of the immune system (e.g., autoimmune diseases), and could also serve to limit the aggressiveness of the immune response [35]. On the other hand, some authors [12,19,36,37] observed reductions of cytotoxic cells and an increase in the CD4/CD8 ratio following folate and vitamin B_12_ deficiency. These observations, however, have not been confirmed in the present study.

The proliferative response in mixed cell cultures is frequently used to estimate “adaptive immunity”. When the proliferative capacity against different mitogens in lymphoid organs was evaluated, we did not observe any change due to the dietary treatment, except for an increase in the spleen lymphoproliferative capacity in response to LPS in the B_12_ deficient/FA supplemented group. Therefore, feeding aged rats with additional folate resulted in an improved response of the spleen to mitogens. Otherwise, it is suggested that vitamins B_6_ and B_12_ deficiencies may induce a decrease in lymphocyte proliferative response against a mitogen [38] and that a dietary folate deficiency may reduce the stimulation of lymphocytes by PHA [39]. It is necessary to emphasize that there are not studies in experimental animal models to validate this hypothesis. Further testing would be of interest to determine some guidelines to determine if FA supplementation may reverse the decline in lymphocyte proliferation when it is associated with a vitamin B_12_ deficiency, and if a deficient state in both vitamins actually decreases the proliferative response.

The cytokines can be modulated by nutrients and their participation is essential in establishing certain mechanisms involved in the development of infectious processes [40]. Therefore, it is necessary to establish whether an inadequate or poor state of FA and vitamin B_12_ may affect the balance between Th1 and Th2 and thus impair the immune response. In this sense, cytokine production was measured in our study not only under spleen cell cultures stimulation with different endotoxins, but also after ConA, LPS and PHA stimulation. IL-1a, IL-1b, IL-4, IL-12, GM-CSF, IFN-γ and TNF-α levels were quantified. These cytokines were selected because they belong to the subset 1 and 2. In light of our results, no significant differences in any cytokine production in unstimulated cells or cells stimulated with ConA, LPS and PHA were observed. It therefore appears that both vitamin B_12_ and FA have no effects on cytokine production in the spleen, and FA supplementation does not improve the cellular immune response when there is a vitamin B_12_ deficiency, but also does not show any negative and/or adverse effect.

## 5. Conclusions

An imbalance in B_12_ and FA alters the NK cytotoxicity and B lymphocyte in aged rats after just a short-term dietary treatment. These effects have not been observed in control or in induced deficiency for both vitamins. The overall balance of folate and B_12_ could be as important as their absolute dietary concentration.
